# Undergraduate Nursing Students' Perceptions of Learning During Clinical Practice When Using a Conceptual Learning Model Grounded in a Caritative Caring Perspective: A Phenomenographic Study

**DOI:** 10.1111/scs.70029

**Published:** 2025-04-24

**Authors:** Maria Koldestam, Gunilla Lindqvist, Anders Broström, Susanne Knutsson

**Affiliations:** ^1^ Department of Accident and Emergency, Region Jönköping County, and Department of Medical and Health Sciences Linköping University Jönköping Sweden; ^2^ Faculty of Health and Life Sciences, Department of Health and Caring Sciences Linnaeus University Växjö Sweden; ^3^ Department of Nursing, School of Health and Welfare Jönköping University Jönköping Sweden; ^4^ Department of Clinical Neurophysiology University Hospital Linköping Sweden; ^5^ Department of Health and Caring Sciences Western Norway University of Applied Sciences Bergen Vestlandet Norway; ^6^ CHILD Research Group Jönköping University Jönköping Sweden

**Keywords:** caritas, clinical practice, conceptual learning models, learning, perceptions, phenomenography, undergraduate nursing students

## Abstract

**Aim:**

To describe the variations in undergraduate nursing students' perceptions of learning during clinical practice when using the conceptual learning model, Model for Improvements in Learning Outcomes (MILO), grounded in a caritative caring perspective.

**Background:**

A conceptual learning model grounded in hermeneutics and a caritative caring perspective addressing ethical values of caring and learning, intertwining didactics, nursing, pathophysiology and medicine to facilitate nursing students' learning during clinical practice and ease challenges in relation to healthcare and supervision was implemented.

**Methods:**

A qualitative descriptive design with a phenomenographic approach was used. Twenty strategically sought undergraduate nursing students in semester six from one university participated (19 women and 1 man aged between 23 and 40 years). Data were collected through semi‐structured individual interviews after the model had been applied in a 7‐week clinical practice course in different departments (surgical, medical and psychiatric/medical rehabilitation care) at 3 hospitals and in 13 municipalities (home care in southern Sweden) and then analysed to identify variations (similarities and differences) in ways of understanding the phenomenon of students' learning using MILO.

**Results:**

Five mutually exclusive descriptive categories of what MILO's concepts and applications had meant for the students' learning emerged; the outcome space was illustrated by the following metaphors: a way to bridge the learning threshold; a way to learn to incorporate the spirit of meaning in caring; a way to learn to put one's soul into something; a way to notice the atmosphere's impact on learning; and a twosome's contradiction in the learning.

**Conclusions:**

Achieving a synthesis of ethical, aesthetical, theoretical and practical knowledge in becoming professional caring nurses was found to be facilitated using MILO. However, the use of peer learning was perceived as contradictory.

## 1 Introduction

Clinical practice is recognised as a vital part of undergraduate nursing education. It is essential to ensure that, when learning and encountering patients during clinical practice in diverse healthcare clinical settings such as hospitals, elderly care, outpatient clinics and home care [1], nursing students are given the prerequisites to achieve competencies [2], the knowledge and skills needed for their future professional role as caring nurses [3]. Broad, advanced knowledge and the individual student's personal development are the basic goals for student learning in higher education [4]. During clinical practice, quality and safety competencies, knowledge and skills in pathophysiology, medicine and nursing [5] must be intertwined with a caring approach [6].

However, learning during clinical practice is complex and several issues are related to this complexity. One issue is the fulfilment of the required minimum time for clinical practice regulated by the EU directives [7]; clinical practice, in terms of time, must comprise half of the students' education and be performed in a clinical setting [8, 9, 10]. Another issue is access to skilled supervisors with time allocated for that task [11]. Reports highlight that there is a lack of clinical work in everyday care among newly graduated nurses [12], and self‐assessed competencies among nurses in their first years of practice show weaknesses in pathophysiology and advanced technical skills and deficiencies in their caring role [13]. A previous study highlight that new graduates need extra support in their nursing roles [14]. Providing competent, caring, person‐centred care and service is a major concern for healthcare organisations worldwide [15, 16], and the need for cooperation between several organisations has been identified [1, 17, 18, 19]. To meet these challenges, education for undergraduate nurses needs to be further developed to include a high standard of clinical practice [1, 20, 21].

In the process of understanding and becoming a nurse [22], a caring relationship with the preceptor is important for the student. An ability to see and analyse a concrete situation is necessary [23] and on a personal level, the carer needs to choose who he/she wants to be [24]. As a result, ethos (i.e., values of responsibility and a sense of at‐homeness) may come into focus [25, 26] with a willingness to care from the heart [26, 27]. Within the tradition of caring science, Eriksson [28] sees caring as the core in nursing; that is, the deepest motive for care is the caritas motive, an act of ethics, grounded in love, mercy and compassion. The mission of the human being is to take responsibility and thereby show respect for human dignity. By understanding, students' knowledge about caring and nursing appropriates [29] and theory thereby becomes natural and concrete in their acts and language [30].

We did not find any other learning model grounded in a caritative caring perspective to facilitate undergraduate nursing students' learning and meet challenges in connection with healthcare and supervision, therefore a conceptual learning model, Model for Improvements in Learning Outcomes (MILO) embracing the basic motive of caring (i.e., love and compassion), was developed [31]. Close collaboration took place between a university and the clinical faculty organisations in a region in southern Sweden between autumn 2015 and spring 2018 to construct and to implement MILO in 3 hospitals (one medium‐sized county hospital and two smaller hospitals) and 13 municipalities in the same region [32]. Conceptual models address the phenomena of interest to a discipline and such a model can be used to establish theoretical values in education [33, 34]. In MILO, the theoretical foundation is grounded in Gadamer's [30] hermeneutically grounded philosophy of knowledge and understanding based on past experiences, openness and context, and the theory of caritative caring [28, 35] which stresses that caring is the core in nursing and that a caring relation and a caring approach are vital. Caring and learning share common fundamental grounds [36, 37] and in MILO, caring and learning are understood as parallel phenomena [38], which involves the application of a reflective, open and compliant approach towards the student [39]. This wholeness in MILO, aiming to facilitate learning, is used to guide the student's learning during clinical practice.

**FIGURE 1:**
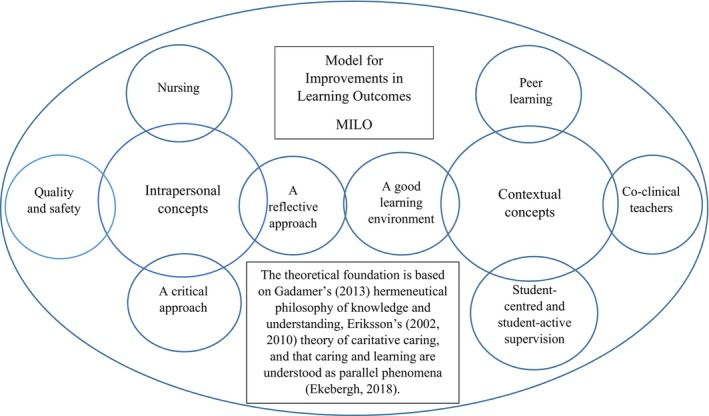
The 8 concepts included in MILO and how they are divided into intrapersonal and contextual concepts as described in Koldestam et al. [31].

MILO consists of eight concepts, four intrapersonal (i.e., the students' own characteristics and abilities important for learning) and four contextual concepts (i.e., environmental concepts) (Figure 1). When applied in MILO (Table 1), these are intertwined with pathophysiology, medicine and skills in nursing, where skills in nursing are seen as a synthesis of ethical, aesthetical, theoretical and practical knowledge [3]. Eriksson and Bergbom [40] describe concepts as windows, which means that new perspectives on caring in praxis can be gained. The concepts in MILO are by supervisors identified as essential for understanding and providing structure to students' learning during clinical practice [31]. The intrapersonal concepts, in comparison with contextual concepts, are valued higher in importance for learning by students in different semesters [41]. From a supervisory perspective, the use of MILO enhanced students' learning when integrating natural and professional care as a result of natural actions and elements contributing to a caritative caring approach in the students [32]. However, the students' perceptions when using MILO during clinical practice are not known. It is important to gain further knowledge about the students' understanding of their learning so that progress can be achieved with the addition of new knowledge and different perspectives. This could contribute to further improvements in the learning model to support students in becoming skilful compassionate caring nurses as well as support supervisors in their tasks, leading to improvements in clinical practice syllabuses and strategies for organisational support. Therefore, the aim of this study was to describe the variations in undergraduate nursing students' perceptions of learning during clinical practice when using the conceptual learning model MILO grounded in a caritative caring perspective.

**TABLE 1:** A description of the application of the 4 intrapersonal and 4 contextual concepts.

Intrapersonal concepts			
Nursing	A reflective approach	A critical approach	Quality and safety
A checklist containing nursing/practical skills Learning activities based on nursing actions/practical skills	A diary based on students own private reflections A reflection sheet: students' daily written reflections and supervisors' responses Reflection seminars: use of patient stories, supervisors' use of open‐ended questions about the students' experience, a questioning approach with use of reflection according to ‘head, heart and hand’^a^	Use of clinical reasoning Use of the feedback model ‘Debriefing with good judgement’^b^.	Learning activities based on quality and safety

Contextual concepts			
Peer learning	Co‐clinical teachers	Student‐centred and student‐active supervision	A good learning environment
Learning activities that should be performed with peers based on nursing/practical skills and quality and safety	Visits to students and supervisors on the wards, answering questions, reflecting with students, supporting the students' performance of learning activities, supporting the supervisors in their reflections with the students and in their supervision of students, supporting the supervisors' assessment of the students' performance	A PM with concrete suggestions for the supervisor and the students on how to outline and plan the weeks in clinical practice; comprises content and progression for maximum learning outcomes	A caring approach: individual meetings between supervisor and students characterised by openness and compliance Affirmation: supervisors should pay attention, listen and give feedback to the students about what is going to happen; using a questioning approach with a desire to see the uniqueness in every situation Straightforward communication; trustworthy and honest communication

^a^ (Eriksson K et al. Den trojanska hästen: evidentbaserat vårdande och vårdarbete ur ett vårdvetenskapligt perspektiv [The Trojan Horse: Evidence‐Based Caring and Nursing from a Caring Science Perspective]. Vasa: Åbo Akademi: 1999).

^b^ (Rudolph JW et al. There's no such things as “non‐judgemental” debriefing: a theory and a method for debriefing with good judgement. Simul Healthc. 2006:1 (1):49–55).

## 2 Methods

### 2.1 Design

A qualitative descriptive design [42] with a phenomenographic approach [43, 44] was used in this study. The SRQR [45] reporting guideline was used to enhance the quality and transparency of the data. When using phenomenography, originally developed within educational research, the intention is to capture, categorise and describe an aspect of the world as it appears to a person (i.e., the second‐order perspective) instead of describing things as they are (i.e., the first‐order perspective). The second‐order perspective represents variations, that is similarities and differences in ways of understanding. When an analysis is performed in a phenomenographic study, the composition of categories and the descriptions and their relationships represent distinct aspects of the phenomenon that is being explored, and these constitute the outcome space [44, 46]. At a collective level, the categories of descriptions in this study thereby express a pattern in the qualitatively different ways the phenomenon of ‘nursing students' learning’ is understood within a group of informants in relation to the MILO learning model.

### 2.2 Preunderstanding

The data were analysed keeping in mind the ontological distinction between the true world and the human subjectively perceived world in accordance with phenomenography [47]. It must be noted that a researcher and interpreter of a text always have a history and stand as part of a tradition. The researchers of this study acknowledge the values and traditions of a caritative caring science perspective. When engaged in this practice, a critical approach to own prejudices and pre‐understanding, as well as a detailed articulation is needed.

### 2.3 Settings and Participants

**FIGURE 2:**
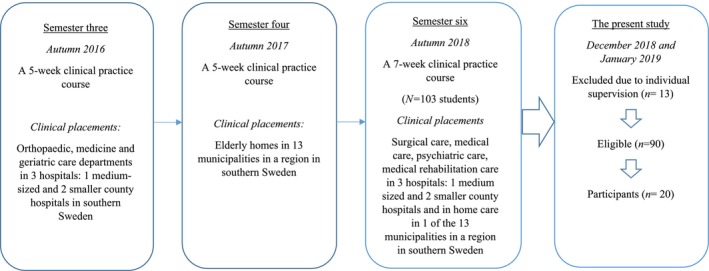
A flowchart of the semesters in the students' education when MILO was applied, together with a description of the participants and the time allocated for the present study.

The study was conducted in autumn 2018 in southern Sweden where the study setting involved the nursing programme at the university, the clinical practice course and the clinical practice placements. MILO was applied for the first time during one 7‐week clinical practice course as part of a theoretical course worth 15.0 higher education credits, conducted on two occasions in semester six of a 3‐year nursing programme which comprises both theoretical and clinical parts, approximately 50% each. The students in the course (*N* = 103) had previously also been supervised and had their learning structured using MILO during clinical practice in semesters 3 (5 weeks) and 4 (5 weeks) (Figure 2). The intended learning outcomes in the course syllabus focused primarily on patients' needs in relation to complex caring situations such as multi‐morbidity and disability, and the learning outcomes connected to the clinical practice course involved students' knowledge, skills, and abilities related to this.

All of the students were orally informed about the study in connection with ordinary teaching/seminars at the university by the first author, who had not had any contact with the students during their clinical practice. A strategic selection of the participants was sought to include a variety of demographic variables and experiences from numerous clinical practice placements in line with the tradition when using the phenomenographic approach [43, 44]. Inclusion criteria were students who had reached the learning outcomes in the course during clinical practice. Exclusion criteria were students who, for some reason, had performed their clinical practice within the course for a second time because they then had been supervised individually, and thereby peer learning was not used in accordance with MILO. After exclusion, 90 students were eligible and potential participants. One reminder about the study was given orally by three regular teachers not involved in the study or the students' clinical practice, and by e‐mails sent from the first author. Twenty students (Table 2) chose to participate, and they were sent a detailed information letter about the study by e‐mail.

**TABLE 2:** Demographical data for the undergraduate nursing students (*N* = 20).

Variable	Distribution
Gender, *n* (%)	
Women	19 (95)
Men	1 (5)
Age, *n* (%)	
23–29 years	16 (80)
30–39 years	4 (15)
40 years	1 (5)
Mean age, years (SD)	27 (4.8)
Previous professional care experience, *n* (%)	
2 months–1 year	6 (30)
2–5 years	8 (40)
6–10 years	5 (25)
11–15 years	1 (5)
Distribution for clinical practice placements, *n* (%)	
Medium‐sized county hospital	11 (55)
Smaller county hospital	8 (40)
Municipality care (community care/home care)	1 (5)
Distribution for clinical practice placements, *n* (%)	
Surgical care (surgery, orthopaedic, urology, gynaecology, oncology) departments	7 (35)
Medical care (medicine, emergency, geriatric, infection) departments	9 (45)
Psychiatric care/medical rehabilitation care departments	3 (15)
Home care	1 (5)

### 2.4 Data Collection

Data were collected through individual semi‐structured interviews [48] conducted by the first author between December 2018 and January 2019. The interview guide was developed with the intention to include questions about all the concepts in MILO. A pilot interview, used in the present study, was conducted to become familiar with the modes of questioning [48] and to assess the interview questions. After dialogue between the first and last author, no changes were made to the questions. The interviews began with an open question: ‘Please tell me what you perceive that the learning model MILO has meant for your learning during clinical practice?’ If the student did not mention all of the eight concepts in MILO, questions were asked about the student's perceptions of the separate concepts in MILO and their application such as “Please tell me what you perceive peer learning has meant for your learning during clinical practice?” Some probing [49] follow‐up questions were asked to clarify some of the statements and achieve a deeper understanding: “How do mean?” “Can you describe some more?” The interviews were held in a calm environment at a time and in a place chosen by the individual student; 2 took place in separate hospitals and 18 in a room at the university's research faculty. Each interview was audio recorded and lasted between 23 and 51 min (average, 36 min). The interviews were then transcribed verbatim by a transcriber not involved in the study.

### 2.5 Data Analysis

The data analysis was performed by the first author using the seven‐step analysis method of Larsson and Holmström [50]. First, the transcribed interviews were read in their entirety; second, the text was read repeatedly; third, the text was marked when the interviewees answered the questions based on the structural (i.e., what) and referential (i.e., how) aspect of the phenomenon, that is, learning related to the use of MILO, and predominant ways of understanding were identified (Table 3). Fourth, after examining similarities and differences, the meaning units that displayed coherence were grouped in categories. Categories of descriptions (i.e., abstractions) were formulated and quotations demonstrating the connectedness between them and the participants' statements were selected. In the fifth step, non‐dominant, that is, other ways of understanding of the phenomenon were identified (Table 3). As a result of the interaction between the separate steps, every single category of description displays uniqueness. In the sixth step, based on the descriptive categories, a structure in the outcome space [44, 46] was created and in the seventh and final step, each category of description was allocated a metaphor. All four authors participated in the analysis process and scrutinised the categories until consensus was reached.

**TABLE 3:** A description of predominating (++) and non‐dominating (+) ways of understanding among the participants (*n* = 20).

Participant	Metaphors				
	A way to bridge the learning threshold	A way to learn to incorporate the spirit of meaning in caring	A way to learn to put one's soul into something	A way to notice atmosphere's impact on learning	A twosomes contradiction in the learning
1	+	+	+	++	
2	++	+	+	+	+
3	++	+	+	+	+
4	++	+	+	+	+
5	++	+	+	+	+
6	+	+	+	++	+
7	+	+	++	+	
8	++				
9	+	++		+	+
10	+	+	+	++	+
11	++	+		+	+
12	+	+	+	++	+
13	++	+	+	+	+
14	++	+	+	+	+
15	+	+	+	++	
16	+	++	+	+	+
17	+		++	+	+
18	++	+	+	+	+
19	+		++	+	+
20	+	+	+	++	+

## 3 Findings

The following metaphors emerged from the data analysis: a way to bridge the learning threshold; a way to learn to incorporate the spirit of meaning in caring; a way to learn to put one's soul into something; a way to notice the atmosphere's impact on learning; and a twosome contradiction in learning. These metaphors represent the students' 5 ways of understanding what MILO has meant for their learning during clinical practice. Anonymized quotations illustrating the findings in each of the descriptive categories are presented in Table 4.

**TABLE 4:** A description of the anonymised quotations illustrating the findings in each of the descriptive categories.

Descriptive category	Quotes
A way to bridge the learning threshold	‘But, then, the supervisors were very attentive…if I had a patient who had undergone some kind of procedure, they asked me: What do you think might happen after this procedure? And if the medication hadn't worked, what should you do then? So this was very important for me…I mean, otherwise, I would not have known if it had had any effect. Did I do it the right way? Did I do it the wrong way? Should I have done it any other way? Hmm…’ (Interview 16)
A way to learn to incorporate the spirit of meaning in caring	‘But I think that we have been able to follow the patient…this means that you get the whole picture…and then, also that you get how complex a situation is…or how complex a situation can become. And…well…there are so many things that you have to understand…and it feels like…well…you quickly learn to identify the needs that exist and what to do to meet the needs.’ (Interview 14)
A way to learn to put one's soul into something	‘You learn a lot. And it is good that there is a bit of stress put on you too…you learn more. Well, you become…you get it when there are challenges. You reflect more…you are really driven; you feel that you want to learn more. I need to learn more…to bring forward what you already know and show what you can actually do.’ (Interview 4)
A way to notice the atmosphere's impact on learning	‘So I could…what is it called…openly… describe a situation without feeling that I shouldn't speak up. You could in a way be a bit private but also develop your professional approach. For example: you are here as a student, but you are also here as a person. What is your previous experience? How does it affect your learning?’ (Interview 10)
A twosomes contradiction in the learning	‘So I think…the idea of peer learning is good. And I think as I have said that it is good to be in a twosome in the first 2 weeks. But then, you should be separated more and more because you need to take care of your own patients. But it is difficult when you have only one supervisor and she asks you questions at the same time. It gets a bit messy. And I felt that our supervisor also found it hard, because she didn't know what to focus on.’ (Interview 5)

### 3.1 A Way to Bridge the Learning Threshold

The students' perceptions focused on the idea that MILO provided attentiveness to what was important when learning nursing. The students described that the use of the applications in MILO, such as the learning activities, different documents, open reflection, when they followed the patients' paths in care and the supervisors' use of the probing questions, had helped them to gain and internalise theoretical knowledge. The use of the applications had also offered different learning approaches and this meant that space for learning was created. The students perceived that the need to know about pathophysiology and the patient's medical problems became clear and the reason for considering the individual patient's needs as a unique human being became visible. Thus, critical awareness and clinical reasoning developed. Concepts in caring science to be used in praxis (i.e., for caring in nursing), such as what is included in a caring relationship, emerged. The use of MILO was helpful when comprehending the broad scope in nursing.

### 3.2 A Way to Learn to Incorporate the Spirit of Meaning in Caring

Learning related to MILO meant that the patient's needs came into focus. The students' perceptions were that when taking care of their “own” patients, as suggested in the PM outlining the content and progression in the coming weeks, they were able to see the patient's needs. The students' perceptions, using this expression, were that they got close to the patient, and this contributed to a change in perspectives, into an understanding of the patient as a whole. When the complexity of caring became noticeable, a deeper understanding developed of how nursing actions affect the patient as a human being. The use of open reflection and listening to other fellow students during reflection seminars led to emotions and challenged the student to consider values, ethical dilemmas and risks in patient care. It also provided knowledge of how to handle new situations and how to give support and comfort to others. The students learned about caring values and the ethical foundations of nursing, and those became more incorporated in their body when using MILO.

### 3.3 A Way to Learn to Put One's Soul Into Something

The students' focus is on taking responsibility to be active, engaged and committed in learning when caring for the patient. The use of MILO allowed them to stretch themselves to reach independence, develop critical awareness of one self and set individual goals in learning. Their own strengths and weaknesses became clear when learning was challenged. MILO increased the students' motivation to actively learn more and review previous knowledge. The students also became more engaged with the patients. The students perceived that they became obliged to learn more and, using MILO, they needed to plan their work and decide what to focus on. They became dedicated to a cause, to an activity. Using the reflection sheet was perceived as helpful for remembering important things and the students described that they became engaged and active in learning.

### 3.4 A Way to Notice the Atmosphere's Impact on Learning

The students' perceptions were directed towards the ethical culture when learning. The students were strengthened when approached with kindness, openness, trust and responsiveness, which they felt were a prerequisite for their progression in learning. Uncertainty arose if MILO was not used in accordance with the plan, such as the use of peer learning or its theoretical grounds, when they or the supervisors did not fully comprehend the documents, found them overwhelming or when supervisors and co‐clinical teachers seemed uninformed. This led to hindered, unstructured learning, causing feelings of stress and loneliness. On the other hand, when supervisors and co‐clinical teachers were attentive to them, a sense of belonging developed, described as a feeling of being included and valuable. Being approached as an individual despite peer learning was essential.

### 3.5 A Twosomes Contradiction in the Learning

The perceptions of learning focused on peer learning, and the use of peer learning was to a great extent perceived to be dependent on good friendship among the peers. Being in a twosome provided confidence when the students interacted with patients on the wards at the beginning of their clinical practice but did not offer enough challenge at the end when they wanted opportunities to perform more challenging tasks on their own. Peer learning was perceived to contribute to independence from supervisors, but it also contributed to competition between the students in terms of attention from the supervisor, but mostly about opportunities to perform practical skills, relying on generosity between the students. Peer learning generated a contradictive perspective when in twosomes; this was both positive and negative at the same time.

### 3.6 The Outcome Space

It was possible to identify the individual student's predominant way of understanding learning in relation to MILO (Table 3), and on a collective descriptive level, the 5 ways of understanding the phenomenon “nursing students' learning” what MILO meant for learning for all involved constitute the outcome space of the phenomenographic analysis. Attentiveness to what was important to learn developed. Students were enabled to see the patient's needs, that is, to incorporate a holistic view of the patient. The students' responsibility to learn developed; they became dedicated, facilitated by increased motivation for learning. Students became aware that the atmosphere was a prerequisite for learning. Contradictive elements, addressed to peer learning. The 5 ways of understanding the phenomenon of inquiry are relatively complex [46] and in this study, similar to another phenomenographic study [51], no hierarchical structure, which could be the last step in a phenomenographic analysis defining a structural relation between the categories [50], could be identified.

## 4 Discussion

This study sought to describe the variations among undergraduate nursing students' perceptions of learning during clinical practice when using a caritative caring conceptual learning model. The 5 mutually exclusive ways of understanding, described by metaphors [50], relate to the hermeneutic unit constituting MILO [31]. Using metaphors in research means that abstract and complex phenomena may be understood when articulated in the light of something else [52] as an interpretive framework [53].

In this study, the students' perceptions were that MILO provided attentiveness to what they needed to learn during clinical practice. Open reflection and the supervisors' use of probing questions were perceived by the students to contribute to their awareness of the reason for learning. According to Eriksson [3], knowledge in nursing can be understood as a synthesis of ethical, aesthetical, theoretical and practical knowledge, and education should serve as a guide in the student's development when learning. In nursing, it is essential to respond appropriately to one's professional responsibilities, and it is important to acquire the knowledge, skill, energy, motivation, judgement and experience—competencies that are humanised by compassion [54]. When learning nursing, a threshold (i.e., a movement between an intertwined holistic picture and loose fragments of knowledge) needs to be crossed to reach an in‐depth understanding [55, 56]. Sandvik et al. [55] suggest that the time needed to pass the threshold may be short, like a sudden awakening, but students often need longer periods. Lassenius [57] highlights that in the students' exploration of the meaning of caring in praxis, both time and insight are essential to discover hidden factors.

Using MILO, caring and learning are viewed as parallel phenomena [38], based on Eriksson's [36] and Eriksson and Matilainen's [37] thoughts about learning and nursing sharing common fundamental grounds, including theoretical concepts. In our study, students became aware of the concepts of caring science when using the applications in MILO. A recent study by Helou et al. [58] highlighted the importance of integrating theoretical concepts and theories relevant to praxis when bridging the theory‐practice gap, and these should preferably be integrated with the students' skill acquisition opportunities. In our study, the students perceived that the applications highlighted what they needed to accomplish when learning. They perceived that the applications together with MILO's theoretical grounds, helped them reach a deeper understanding of what nursing and caring in nursing are about, and thus served as a way to bridge the threshold; knowledge about this could be helpful in the design of clinical practice.

In our study, the students' perceptions focused on the fact that MILO offered understanding of the patient as a human being. This was related to the possibility of taking care of “own” patients as well as the use of open reflections, applications and approaches in accordance with MILO. When students experience patients' reactions when caring for them, feelings of compassion and courage can be a source of energy, which helps them to focus on the patient [59] and feelings of responsibility may develop [27]. Eriksson [23] claimed that absolute presence and insight in a situation with a desire to understand are needed and crucial if the knowledge is to be beneficial to the patient. Our language, words and concepts are necessary stipulations to absorb reality, and together with the use of reflection, understanding can be reached [26, 30]. In our study, the change in the students' perspectives when confirming (i.e., dignifying) [28] the patient's needs, a way to learn to incorporate the spirit of meaning in caring, may be understood as a striving towards an ideal based on ontological values, an ethos [3, 60, 61] where MILO served as an ethical eye opener. Knowledge gained becomes visible through arête, actions performed with joy and with a natural curiosity to learn [40, 62]. The concept of phronesis (practical wisdom) can be seen in concrete actions [63]. Good judgement means that evidence, our seeing, knowing and insights are decisive when caring [40].

In this study, increased motivation to learn was perceived by the students when using MILO. Awareness of one's self and dedication developed in terms of acknowledgement of knowledge gained, but also lack of knowledge. Reaching independence was perceived as essential. Reflecting about thinking may contribute to new perspectives [64] and the use of MILO as a way to learn to put one's soul into something is understood to be initiated by the different activities when using MILO, which challenge the students to actively engage themselves in their learning and in the patient, both theoretically and practically. To be there, to be engaged, to see, listen to, to confirm the patient in a caring encounter is essential for the patient's well‐being, and self‐awareness is described as being essential [65].

In this study, using MILO, ethical culture values when learning engaged the students. The students' perceptions were focused primarily on the supervisors' and co‐clinical teachers' approaches towards them and the resulting emotional state that developed within them, due to both positive and negative encounters. Lack of attention towards them as individuals led to feelings of insecurity, but supervisors' compliance and keen eye developed feelings of trust, confidence and a sense of progression in learning. The students became aware of the ethical values that were important for their learning and MILO thus became a way to notice the learning atmosphere. Säfström [66] argued that progression needs to be understood in relation to a perspective of learning and it is in dialogue with a learning theory that progression can be specified. A bearing of a caritative caring culture related to MILO and its necessity for learning has been discussed previously by Koldestam et al. [41]. The impact that a caring learning environment and supportive supervision has on learning is also recognised by Nyqvist et al. [67] and mutual trust between students and supervisors is described as essential for students to expand their knowledge during clinical practice. To be recognised, seen and heard, thus dignified, facilitates professional development when learning nursing [68].

When using MILO, the students perceived contradictions in learning, and peer learning was at the centre of these perceptions. These contradictions were related to aspects of how peer learning should be managed and when peer learning should be applied; mostly, it concerned the students' well‐being when interacting with their peers. The students also perceived the possibilities of reaching independence using MILO; students' ability to reach a high level of independence has been highlighted in EU directives as essential [4]. A recent study by Koldestam et al. [41] showed that students who used MILO in early semesters valued peer learning more than students who used MILO in later semesters. However, in this study, the students' perceptions covered both positive and negative aspects using peer learning, but no connections to clinical placements or semesters could be identified because the students' perceptions fluctuated. This could be understood as learning, using MILO, is a twosome contradiction, because aspects of the phenomenon were both negative and positive at the same time.

### 4.1 Methodological Considerations

A sample of 20 respondents, as used in this study, is usually enough [69] to capture variation in the participants' backgrounds and awareness of how a specific phenomenon is understood using the phenomenographic approach [44]. Fewer men than women, common in nursing programs in Sweden, participated in the course, and only one chose to participate in the study which means that a comprehensive understanding of male students' perceptions is lacking. Another limitation might be that only one student had performed clinical practice in community/home care.

With a desire to achieve negotiated consensus [70] to strengthen the trustworthiness [42] of the study, co‐assessment was performed by the co‐authors in several rounds during the analysis process. The credibility [71] of the study is strengthened by the researchers' self‐awareness of their own pre‐understanding, the one performing the interviews with the students was not responsible for the MILO course or in an active way met the students involved in the course, the description of the interview questions, the fact that the analyses were performed shortly after the students had finished their clinical placement, the visibility of the quotes chosen, and the relationship between the categories and the empirical data [72]. The use of semi‐structured interviews offered flexibility [73] in the interview situations, which enabled the participants to elaborate on their perceptions of each concept in MILO and MILO as a whole in line with the phenomenographic approach [44]. Empathic listening [74] has guided the data collection process, allowing the individual students to open up about their life‐world. The quotes used in the study have been chosen carefully so that none of the students can be identified. Challenging situations in connection with clinical practice were sometimes recounted during the interviews. Students showing a need for extra care were offered time to reflect after the interviews.

## 5 Conclusions

Five ways of understanding the meaning of learning using MILO, in whole or in part, were revealed. The students developed attentiveness and became aware of the reason for learning. Hence, MILO served to bridge the learning threshold, and the students developed a holistic view of the patient. Increased motivation to learn and commitment evolved, and the students developed responsibility for their own learning. The atmosphere, including ethical values when learning, was noticed by the students. Achieving a synthesis of ethical, aesthetical, theoretical and practical knowledge in becoming a professional caring nurse could thus be understood to be facilitated using MILO. This knowledge could be valuable for university and clinical faculties when designing clinical practice. However, aspects of peer learning contributed to contradictive perceptions of learning. This needs to be looked at more closely in future studies.

## Author Contributions

M.K., A.B., and S.K. designed the study. M.K. performed the interviews and data analysis. All authors were involved in the interpretation of the data. M.K. was responsible for drafting the manuscript. All authors contributed to critical revisions.

## Ethics Statement

The research was reviewed by the Boards of Ethics Committee in Linköping, Sweden (ref. 2018/490‐31) and approval for the study was obtained from the Dean of the university. The principles according to the World Medical Association [75] and the ethical guidelines according to the International Council of Nurses [76] were considered carefully; oral and written information was given to the participants about the purpose of the study, that participation was voluntary, could be withdrawn at any time, and written informed consent was obtained before the interviews. Only the authors of the study have had access to the data. The interviews were performed after the students had completed their clinical practice to ensure no connection with assessments or grades and thereby reduce the risk of dependency. The first author who performed the interviews was the only person who knew the identity of the participants.

## Conflicts of Interest

The authors declare no conflicts of interest.

## Data Availability

The data that support the findings of this study are available on request from the corresponding author. The data are not publicly available due to privacy or ethical restrictions.
